# Biomarkers for Optimization and Personalization of Anti-TNFs in Pediatric Inflammatory Bowel Disease

**DOI:** 10.3390/pharmaceutics13111786

**Published:** 2021-10-26

**Authors:** Sara Salvador-Martín, Alejandra Melgarejo-Ortuño, Luis A. López-Fernández

**Affiliations:** 1 Servicio de Farmacia, Instituto de Investigación Sanitaria Gregorio Marañón, Hospital General Universitario Gregorio Marañón, 28007 Madrid, Spain; sara.salvador@iisgm.com (S.S.-M.); Alejandra.melgarejo@salud.madrid.org (A.M.-O.); 2 Spanish Clinical Research Network (SCReN), 28040 Madrid, Spain

**Keywords:** biomarkers, pharmacogenomics, personalized medicine, inflammatory bowel disease, infliximab, adalimumab, Crohn’s disease, ulcerative colitis

## Abstract

The use of biological drugs has improved outcomes in pediatric inflammatory bowel disease (IBD). Prediction of the response to biological drugs would be extremely useful in IBD, and even more so in children, who are still growing physically and psychologically. Specific clinical, biochemical, and genetic parameters are considered predictive of response to biological drugs, although few studies have been carried out in children with IBD. In this review, we present current evidence on biological treatments used in pediatric IBD and the available biomarkers of response. We examine demographics, clinical characteristics, biomarkers (genetic, genomic, and cellular), and microbiota.

## 1. Introduction

Inflammatory bowel disease (IBD) is a chronic immune-mediated condition that affects the gastrointestinal tract. Almost 25% of cases are diagnosed before the age of 18 years [1], 20% before the age of 10 years [2], and 5% before the age of 6 years [3]. The incidence of the disease in children is increasing [4]. However, most clinical trials in this disease have been carried out in adults, and the results have been extrapolated with minimal changes to determine treatment in children. Pediatric IBD (pIBD) is characterized by various factors, including a more severe phenotype than adult disease [5,6]. Since IBD is a chronic autoimmune disease, patients diagnosed during childhood live longer with the illness and consequently need treatment for longer.

Biological drugs and, more specifically, anti-TNF drugs such as infliximab and adalimumab have proven efficient for treatment of IBD in adults and in children [7]. However, the use of biological drugs differs between children and adults with IBD [8]. For instance, the time between diagnosis and initiation of biological treatment is shorter in children than in adults [9,10]. In addition, not all the biological drugs approved for adult IBD are approved for children. Therefore, treatment with biological drugs should be personalized as much as possible in pIBD in order to avoid early non-response and maximize duration of response in children.

There is an urgent unmet need for predicting response prior to treatment initiation to reduce healthcare costs and avoid unnecessary treatment, allowing a more rational use of resources. While identification of biomarkers for response to biologics in IBD has been a priority in the last 15 years, results have only been partially positive. Unfortunately, most biomarkers have been identified in adults or in populations combining adults and children [11]. Very few studies have been performed exclusively in children with IBD, although the results do point to common biomarkers for both populations and to others that are specific for children. Since susceptibility to IBD differs between the two populations [12,13], there may also be differences in their response to the biological drugs used to treat the disease. Separate searches were performed for each part of the review and once the manuscripts were analyzed, they led to other articles not detected in the searches. Some manuscripts mentioned in the review were included because of a thorough and regular reading of manuscripts over the years on this topic by the authors.

In this review, we summarize clinical, biochemical, genetic, genomic, and cellular biomarkers of response to biological drugs in pIBD.

## 2. Pediatric Inflammatory Bowel Disease. Differences with Adults

### 2.1. Clinical Differences

The natural history of pIBD is characterized by a more severe phenotype than in adults. Regarding extension, intestinal involvement is more relevant in childhood, with more rapid and aggressive disease progression [14,15,16]. A study comparing IBD patients (21,200 adults and 846 children) confirmed this evidence and demonstrated that childhood-onset IBD was associated with an higher risk of immunomodulator use [17]. In children, the most common symptoms include diarrhea with or without blood, abdominal pain, and malnutrition [18].

Approximately 50% of patients experience extraintestinal disorders, which are usually the initial manifestations of the disease [19]. In children, these are associated with more severe disease course [20] and include weight loss, growth failure, late pubertal development, and psychosocial problems [21,22], all of which are particularly relevant in this population. Extraintestinal manifestations are very frequent in pediatric Crohn’s disease (pCD), affecting 10–30% of cases [21,23,24].

Differences in CD and ulcerative colitis (UC) between adults and children have led to the modification of the adult Montreal classification, which has been adapted to the pediatric population, and to the creation of a new classification, the Paris classification, which reflects changes that may occur during childhood and also allows for growth abnormalities, which, as previously mentioned, are very relevant in this population [2,25].

In summary, developing pediatric IBD, an incurable inflammatory intestinal disease that influences growth and puberty in patients at a vulnerable psychosocial age is even more challenging than when it is diagnosed in adults.

### 2.2. Treatment of Pediatric IBD

The goal of the treatment of pediatric IBD is to induce and maintain clinical remission, achieve normal growth, provide optimal quality of life, promote psychological health, and reduce toxicity as much as possible. Additionally, the gold standard of optimal therapy is endoscopic mucosal healing, which makes it possible to modify the natural history of the disease and prevent complications of progressive bowel destruction. In observational adult studies, younger age at onset is repeatedly considered high-risk for poor prognosis, thus underlining the need for a highly effective treatment approach in children [26].

Treatment is selected based on the location, type of disease, severity of symptoms, and the goal of therapy (induction therapy or maintenance of remission). The pharmacological arsenal for pIBD treatment includes anti-inflammatory drugs such as aminosalicylates, corticosteroids, and immunomodulatory drugs (for example, thiopurines and methotrexate), which are used as maintenance therapy, and biologic drugs, which are used for induction and maintenance of remission. The doses and treatment guidelines for biologic drugs are very similar to those of adults, even though the metabolism and immune system of children may differ from those of adults [27,28,29].

The introduction of monoclonal antibodies against tumor necrosis factor (anti-TNF) revolutionized the treatment of IBD. Infliximab and adalimumab are the two anti-TNF agents approved by the United States Food and Drug Administration (FDA) and the European Medicines Agency for use in children, although adalimumab is not approved in perianal pCD [30]. Infliximab is administered as an intravenous infusion and adalimumab is administered subcutaneously for induction and maintenance therapy. Studies have shown that early use of anti-TNF drugs in children with CD is associated with increased rates of remission and mucosal healing, as well as with modest improvement in linear growth [15,30,31,32].

Recently, the European Crohn’s and Colitis Organization (ECCO) and the Paediatric IBD Porto group of the European Society of Paediatric Gastroenterology, Hepatology, and Nutrition (ESPGHAN) updated their recommendations for the medical management of pCD [33]. According to their guidelines, patients with perianal disease, penetrating type, or severe growth retardation should be considered for up-front anti-TNF treatment in combination with an immunomodulator.

Advances in the understanding of the etiology and pathogenesis of IBD in recent years have led to the development of new drugs based on inhibition of immune cells [34] or inhibition of cytokine signaling [35,36,37]. New categories of biologic drugs that have been shown to be effective and safe in adults are the new horizon for IBD treatment in children [30]. Some biological drugs that are currently approved in adults, such as vedolizumab or ustekinumab, are used off-label in children when treatment with infliximab or adalimumab fails [38].

Despite advances in medical treatment, surgery may still be warranted in refractory pIBD [26]. In pCD, the median time to first surgery is longer than in patients who debut in adulthood, although the need for surgery in pUC is earlier than in adults. Consequently, the risk of surgical resection before the age of 30 years is higher in children than in adults [39].

## 3. Clinical and Biochemical Biomarkers of Response to Anti-TNFs in pIBD

Anti-TNFα drugs have proven to be effective and safe for pIBD [14], although approximately one third of patients who initially respond to anti-TNF therapy lose that response over time [40,41]; and while various clinical and biochemical characteristics predict response to anti-TNF therapy, these are mainly based on studies in adult populations [42]. The characteristics include disease-related factors (such as disease phenotype, behavior, location, and severity), biochemical parameters (such as C-reactive protein, fecal calprotectin, and albumin levels) and drug-related characteristics (such as pharmacokinetic, pharmacodynamic, and immunogenic factors) [43,44,45,46,47]. The ECCO-ESPGHAN guideline update on management of CD in children recommends monitoring of fecal calprotectin or small bowel imaging as the best markers of treatment response [33].

The PANTS study is one of the few studies to evaluate the response to anti-TNFs in a population including children and adolescents over 6 years of age, although to date, no subanalysis of pediatric patients has been performed. Obesity, smoking, low albumin concentrations, higher baseline markers of disease activity, and development of immunogenicity were associated with low drug concentrations during induction, resulting in non-remission at week 54 after initiation of anti-TNF treatment [48].

The level of anti-TNF agent immediately before the following administration, known as the trough level, is increasingly used as a non-invasive biomarker. It is well known that serum levels of infliximab and adalimumab correlate with treatment response in patients with IBD [49,50] and pIBD [51,52,53,54]. Furthermore, these levels are associated with histological and endoscopic disease remission in both populations [55,56,57,58].

The therapeutic range of these drugs varies considerably, especially in pIBD. Most guidelines indicate that to achieve clinical remission of IBD, infliximab and adalimumab concentrations in the range of 3–7 and 5–12 μg/mL, respectively, are considered adequate [43,59,60,61,62]. The therapeutic ranges of both anti-TNF drugs may vary depending on the disease phenotype or on the treatment goals [48,58,63]. Further studies are needed to define optimal levels.

Anti-TNF drugs are antibodies against TNF that can induce the immune response and generate anti-drug antibodies (ADAs). ADAs bind to the anti-TNF drug, thus reducing free functional drug levels, neutralizing the therapeutic effect, and resulting in a loss of response [64]. ADA levels inversely correlate with drug levels and treatment response in adults [45,65], as well as in children [66,67,68,69].

For this reason, therapeutic drug monitoring (TDM) has been proposed as a means of optimizing biological therapies in both adults [70,71,72,73] and children [52,74,75,76] with IBD. This approach appears to be more advantageous in pediatric patients, since fluctuations in pharmacokinetic variables tend to be more pronounced in children than in adults, possibly owing to physiological differences, such as volume of distribution, and immaturity of enzyme systems and of clearance mechanisms [52]. In fact, Jongsma MME et al. reported that, over one year of treatment with infliximab, patients under 10 years of age require a more intensive treatment regimen than older patients and that these patients are more likely to develop antibodies to infliximab [77].

Data on the optimal timing of TDM are conflicting, since some professionals use reactive monitoring, i.e., measuring drug levels in the case of loss of response, whereas others use proactive monitoring, i.e., measuring them at preset time points [78]. Proactive monitoring has been shown to achieve clinical improvement and endoscopic remission in IBD patients treated with anti-TNFs [79,80,81], as well as in children [82,83]. However, this issue is quite controversial and, in fact, the recommendations form the ECCO for adults are indecisive [33].

The current recommendation in pIBD is to measure drug levels and ADA titers after the induction period, even though studies in this population are insufficient and data are conflicting [30,54,84]. The use of TDM in pIBD is increasing in clinical practice, and efficacy similar to that of adults has been demonstrated in children, with loss of response to anti-TNF therapy [51].

Considering the high cost and potentially severe side effects of anti-TNF biologics, the identification of underlying factors involved in the individual responses is sorely needed. The usefulness of TDM is therefore limited, as monitoring helps physicians to modify the existing treatment by adjusting the dose of the biological drug and/or the frequency of administration. However, to choose the best biological drug and the best starting dose, other types of biomarkers are needed. Moreover, these new biomarkers should be inexpensive and easy to implement in clinical routine, which is not always simple.

## 4. Genomic Biomarkers of Response to Anti-TNFs in pIBD

Pharmacogenomics may play an important role in predicting response, mainly before initiation of anti-TNF treatment in pIBD. Genetic variants and gene expression could be useful markers for predicting response to biological drugs in children with IBD. Since pediatric patients will have to live longer with the disease and will therefore need treatment for longer, identification of pharmacogenomic biomarkers with the aim of personalizing treatment is especially important in this population.

### 4.1. Genetic Variants

The genetics of pIBD differs from that of adult IBD, thus highlighting the relevance of finding specific biomarkers for children [85]. Several single-nucleotide polymorphisms (SNPs) have been associated with the response to anti-TNF drugs in adult patients with CD, UC, or IBD. The most relevant SNPs are located at various sites: in genes involved in the NF-kB signaling pathway activated through *TLR2*, *TLR4*, *TLR5*, *TLR9*, *LY96*, *CD14*, *MAP3K14*, *NFKBIA*, and *NFKB1*; in genes of the TNF signaling pathway activated through *TNF*, *TNFRSF1A*, *TNFRSF1B*, and *TNFAIP3* and other cytokines and their receptors regulated by this pathway, such as *IL1B*, *IL1RN*, *IL6*, *IL10*, *IL17A*, and *IFN*; in other genes involved in the regulation of inflammation, for instance, *IL4R*, *IL6R*, *IL23R*, *TGFB1*, *PTPN22*, *PPARG*, and *NLRP3*; and in genes involved in autophagy and apoptosis, such as *ATG16L1*, *ATG12*, and *ATG5* and *FASLG* and *FCGR3A* [9,44,86,87,88,89,90,91,92,93,94,95,96].

Of note, information on these biomarkers of response to infliximab and adalimumab in pIBD is lacking. Few studies have included children [97], and even fewer have focused only on children [98,99,100,101]. The two main strategies followed are selection of genome-wide association studies (GWAS) [97,98] and selection of SNPs [10,101]. Another approach has been to identify the genetic variants associated with parameters that correlate with a higher probability of response, such as biological drug trough level [100,101].

Dubinsky et al. aimed to find SNPs associated with response to anti-TNF drugs in 94 children with IBD by GWAS in order to develop a predictive model for primary anti-TNFα non-response [98]. The authors found 65 SNPs associated with primary response to infliximab in pIBD with a *p* value < 0.0001 (Table 1). They then tested for predictive models of non-response to infliximab. The best predictive model included diagnosis, pANCA, and the following SNPs: rs2836878 (*BRWD1*), rs975664 (*TACR1*), rs4855535 (*FAM19A4*), and rs6100556 (*PHACTR3)* [98]. The SNP rs2836878 was one of the susceptibility loci for pIBD and had not previously been reported in adults with IBD [102]. This does not indicate that it is not associated with adult IBD, but rather suggests that there may be differences between the two populations, thus highlighting the importance of separating them when studying pharmacogenetic markers. The study by Dubinsky et al. is the largest to try to identify genetic variants associated with response to biological drugs in children with IBD.

Our group recently applied another strategy to analyze response to anti-TNFs in pIBD. We used Kaplan–Meier curves to analyze 21 SNPs in *TLR2*, *TLR4*, *TLR9*, *LY96*, *CD14*, *MMP3K14*, *TNFRSF1A*, *TNFRSF1B*, *TNF*, *TNFAIP3*, *FASLG*, *IL10*, *IL1B*, *IL6*, and *IL17A* in association with long-term response in pediatric patients diagnosed with CD or UC [10]. Using this approach, we identified the polymorphisms rs1800872 (*IL10*), rs2275913 (*IL17A*), rs10499563 (*IL6*), and rs3397 (*TNFRSF1B*) as being associated with response to anti-TNF agents in pCD. None of these SNPs was significantly associated with response to infliximab in adults diagnosed with CD, suggesting that they could be differential biomarkers for response to biological drugs in children [9]. In the same study, the SNP rs11465996 in *LY96* was associated with response to anti-TNF drugs in pUC. This SNP has not yet been studied in adults with UC.

Another strategy used to identify SNPs related to efficacy of biological drugs in pIBD is the use of parameters associated with a higher probability of response, such as biological drug trough level (see above).

This strategy was first successfully tested in adults with CD treated with infliximab [9]. The same approach was subsequently used to identify genetic polymorphisms associated with serum trough levels of infliximab and adalimumab during maintenance therapy in children diagnosed with IBD (Table 1) [100]. Remarkably, patients with rs1816702 CC (*TLR2*) had almost double the serum trough levels of patients with the CT or TT genotypes. This difference was not observed in serum trough infliximab levels. These differences between infliximab and adalimumab could facilitate selection between the drugs according to genotype.

Since anti-drug antibodies are associated with failure of biological drugs in IBD, genetic variants associated with the presence of these antibodies could be useful for predicting response to the drugs. Along these lines, Sazonovs A et al. found that patients carrying the HLA-DQA1*05 allele more frequently had antibodies against anti-TNF agents in adults and in pediatric patients (aged six years or older) with CD. The finding was associated with a worse response to these treatments [97]. Up to 90% of CD patients who received infliximab and carried HLA*DQA1*05 had developed anti-infliximab antibodies by week 54. This is a potential biomarker that should be specifically explored in children.

Consistent with our approach, other authors explored the association of the SNPs rs3936991 (*FCGR3A*) and rs1800629 (*TNF*) with response, serum trough levels, and ADA production in IBD patients treated with infliximab [101]. Curci et al. showed that variant C rs396991 was associated with a poorer clinical response at the end of induction and at 22 and at 52 weeks of treatment with infliximab. In addition, patients with this variant had lower infliximab levels and were more likely to produce ADAs than patients with the wild-type genotype [101].

Unfortunately, none of the SNPs that have been associated with response to anti-TNFs in children have been validated in other studies. More studies are needed to explore the usefulness of these biomarkers. More genetic data are necessary to personalize anti-TNF therapy in patients with pIBD according to type of disease and biological drug to be used.

### 4.2. Biomarkers of Gene Expression

Specific gene expression profiles in the inflamed tissues of adult patients with CD and UC have been associated with response to anti-TNF drugs. The genes identified include *TNFRSF11B*, *STC1*, *PTGS2*, *IL13A2*, *IL11*, *OSM*, *TREM1*, *CCR2*, and *CCL7* [103,104,105,106,107]. Expression of *OSM* and its receptor *OSMR* was recently shown to be higher in the colonic mucosa of adult patients not achieving endoscopic remission [106]. The expression of these genes predicted response to anti-TNF therapy with an area under the curve (AUC) of 73.7%. However, none of these studies focused on the pediatric population. The only gene expression studies carried out in children with IBD to date [99,108] measured gene expression in whole blood, instead of in inflamed tissue (Table 2).

In Salvador-Martín et al., which included 33 children, expression of *SMAD7*, *FCGR1A*, *FCGR1B*, and *GBP1* was found to be a pharmacogenomic biomarker of early response to anti-TNF agents in children with IBD. *SMAD7* expression was decreased in non-responders before initiation of treatment and after two weeks on the treatment [99]. The same authors conducted a transcriptome analysis which revealed 32 genes differentially expressed in children with IBD between responders and non-responders before initiation of treatment with anti-TNFs and 44 genes two weeks later. Of these, *FCGR1A*, *FCGR1B*, and *GBP1* were overexpressed in non-responders after two weeks of treatment with anti-TNFs [108].

Given the invasive nature of biopsies to obtain colon samples, gene expression biomarkers from whole blood could prove advantageous, especially in children. Little is known about this subject in children with IBD. A meta-analysis found genes to be differentially expressed in the blood and colon biopsies of children with UC, thus validating the approach of identifying biomarkers of IBD in blood, although the authors did not explore efficacy of biological drugs [109]. TNF was not upregulated in damaged tissues or in whole blood. Increased expression of receptors in genes such as *TNFRSF1B*, *OSMR*, *IFNAR2*, and *CSF2RA* can explain the lack of increase in their ligand in colonic biopsies. Finally, there is a certain correspondence in gene expression between blood and colon tissue.

Ostrowski et al. identified a group of 15 genes (*ANOS1*, *ANXA3*, *ATP9A*, *CACNA1E*, *COX6B2*, *FCGR1B*, *GALNT14*, *IL18R1*, *ITGB4*, *KLRF1*, *MMP9*, *OPLA*, *PFKFB3*, *S100A12*, and *UTS2R*) in peripheral blood with the potential to discriminate between children with clinically active IBD and healthy donors, but not between adults with IBD and healthy donors [110].

**Table 2:** Gene expression biomarkers associated with disease activity or response to biological drugs in children with IBD.

Gen	Comparison	Time/Tissue	Disease/Treatment	Ref
*SMAD7*	*R vs. NR	0, 2 W/Blood	IBD/Anti-TNFs	[99]
*FCGR1A*	*NR vs. R	2 W/Blood	IBD/Anti-TNFs	[108]
*FCGR1B*	*NR vs. R	2 W/Blood	IBD/Anti-TNFs	[108]
*GBP1*	*NR vs. R	2 W/Blood	IBD/Anti-TNFs	[108]
*ANOS1*	*Active IBD vs. Controls	Blood	IBD	[110]
*ANXA3*	*Active IBD vs. Controls	Blood	IBD	[110]
*ATP9A*	*Active IBD vs. Controls	Blood	IBD	[110]
*CACNA1E*	*Active IBD vs. Controls	Blood	IBD	[110]
*COX6B2*	*Active IBD vs. Controls	Blood	IBD	[110]
*FCGR1B*	*Active IBD vs. Controls	Blood	IBD	[110]
*GALNT14*	*Active IBD vs. Controls	Blood	IBD	[110]
*IL18R1*	*Active IBD vs. Controls	Blood	IBD	[110]
*ITGB4*	*Active IBD vs. Controls	Blood	IBD	[110]
*KLRF1*	*Active IBD vs. Controls	Blood	IBD	[110]
*MMP9*	*Active IBD vs. Controls	Blood	IBD	[110]
*OPLAH*	*Active IBD vs. Controls	Blood	IBD	[110]
*PFKFB3*	*Active IBD vs. Controls	Blood	IBD	[110]
*S100A12*	*Active IBD vs. Controls	Blood	IBD	[110]
*UTS2R*	*Active IBD vs. Controls	Blood	IBD	[110]
*TNFRSF1B*	*UC vs. Controls	Blood/CB	UC	[109]
*OSMR*	*UC vs. Controls	Blood/CB	UC	[109]
*IFNAR2*	*UC vs. Controls	Blood/CB	UC	[109]
*CSFR2A*	*UC vs. Controls	Blood/CB	UC	[109]

NR, non-responders; R, responders; IBD, inflammatory bowel disease; UC, ulcerative colitis; CB, colon biopsies. * Higher levels.

A role as potential biomarkers in IBD has also been proposed for micro RNAs (miRNAs) [111]. One study identified the expression of three miRNAs in whole blood associated with response to prednisone and infliximab in children with pIBD [112]. The list subsequently increased to 11 miRNAs associated with response to anti-TNF treatment, glucocorticoids, or both [113]. The analysis of these 11 genes showed that increased baseline expression, i.e., prior to administration of anti-TNF agents, returned to levels comparable to those of healthy subjects after anti-TNF treatment in eight miRNAs (miR-126, miR-26a, miR-26b, miR-454, miR-146a, miR-146b, miR-320a, and let-7c). Furthermore, five of these miRNAs were also increased in biopsies of inflamed tissue (miR-146a, miR-146b, miR-320a, miR-126, and let-7c) [113]. However, the main limitation of this study was that all the recruited patients responded to treatment.

Another study with miRNAs identified miR-15a as a biomarker of activity in pediatric CD [114]. Since decreased activity directly correlates with response, this molecule could be a potential pharmacogenomic biomarker of biological drugs in children with IBD. More studies are needed to improve our knowledge of miRNAs as biomarkers of pIBD in clinical practice.

A recent search for differentially expressed genes in the anti-TNF response led the authors to suggest that *IL6* levels prior to initiation of infliximab are associated with primary nonresponse (measured at 14 weeks), in addition to body mass index, disease course, and C-reactive protein levels in patients diagnosed with CD [115]. Unfortunately, these authors did not include information about the number of children recruited. It would be interesting to search for a cutoff value of *IL6* as a biomarker or as a predictor of the response to biological therapy in children with IBD. Our group found that the SNP rs10499563 C (*IL6*) was associated with anti-TNF trough levels in adults with maintenance therapy, but not in children [9,100]. More studies are needed to identify the potential role of *IL6* as a biomarker in children, adults, or both.

A study with 913 children with CD found a genetic signature associated with stricturing complications and that early anti-TNF therapy reduces penetrating, but not stricturing, disease complications [116]. This combination makes it possible to personalize anti-TNF treatment in children with CD.

## 5. Other Biomarkers of Response to Anti-TNFs in pIBD

Regulatory T cells (Tregs) play an essential role in the pathogenesis of IBD, in which Treg counts are decreased [117]. Anti-TNF therapy is known to increase the number and function of Tregs in IBD [118]. The study of these cells may help to predict response to anti-TNF agents, because upregulation is not as efficient in non-responders as in responders [119,120].

Few studies have assessed Tregs in children, although preliminary results suggest an effect similar to that observed in adults. Ricardelli et al. showed that FOXP3+ T-cell counts were lower in the mucosal samples of children with active CD than in healthy controls. However, this difference disappeared after the initiation of infliximab [121].

Furthermore, intestinal microbiota may also modulate the immune system and play an acute role in IBD [122]. It has been suggested that defects in Treg function might induce changes in the gut microbiome, leading to loss of tolerance to commensal bacteria [123]. In children, Conte et al. observed higher numbers of mucosa-associated aerobic and facultative anaerobic bacteria in IBD patients than in healthy controls [124]. The authors also observed a decrease in counts of *Bacteroides vulgatus*. A subsequent study differentiating between CD and UC in children showed a decrease in counts of *Faecalibacterium prausnitzii* and an increase in those of *Escherichia coli* in children with CD [125]. However, no differences were found in the composition of microbiota in children with UC, in contrast with findings in adults [125].

Concerning anti-TNF therapy and the gut microbiome in children with IBD, a higher number of multiple short-chain, fatty-acid-producing bacteria has been associated with a sustained response to infliximab in pediatric CD [126]. In addition, infliximab increased the diversity of the gut microbiome, and its composition resembled that of healthy children. These results were recently confirmed in a larger cohort of pediatric CD patients, where bile salt hydrolase-producing bacteria are also enriched after treatment with infliximab [127].

The aforementioned data suggest that Treg count and functionality, as well as the gut microbiome, could act as relevant biomarkers of response to anti-TNFs. However, this observation is restricted to infliximab. More studies are necessary to validate these biomarkers and to find new ones associated with the different biological drugs used in pIBD.

The list of factors thought to affect the efficacy of anti-TNFs is growing. It was recently reported that vitamin D deficiency was associated with a higher risk of early discontinuation of anti-TNF therapy (14.5% vs. 0%) in children with IBD [128].

## 6. Conclusions and Perspectives

In this review, we present clinical, biochemical, genetic, genomic, and cellular biomarkers of response to anti-TNF therapy in pIBD and discuss their differences with adults. We also discuss the role of the microbiome in this disease. Some markers, such as fecal calprotectin, C-reactive protein, and serum trough anti-TNF level are supported by their utility and strong clinical evidence and are useful in current clinical practice. There are currently no predictive biomarkers, prior to treatment initiation, that can be used to guide the personalization of biologic therapy in pIBD. Genomic biomarkers need to be validated in larger cohorts of patients before they can be applied in clinical practice. Some of them are very promising and, if confirmed, could be useful in real practice in a few years.

Several reviews focusing on children with IBD also address prediction of response to anti-TNF agents. However, ours is the only one to add studies specifically performed in children diagnosed with IBD and to focus on biomarkers of response to biological drugs. Research should also be extended to other biologic drugs in IBD that are used off-label in children, such as vedolizumab and ustekinumab. Currently no clear biomarkers have been identified for these drugs in children with pIBD.

Nevertheless, regardless of the reason for the differences observed, the identification of biomarkers of response to biological drugs in pediatric IBD could help to personalize therapy and prolong the useful life of existing treatments, while we wait for new drugs to be developed. Although it is clear that the basis for personalized medicine is not yet available and remains an unmet need in daily clinical practice, the identification of response biomarkers to anti-TNF drugs could improve future treatment in pediatric patients with this chronic disease.

## Figures and Tables

**Table 1:** Genetic variants associated with response or factors related to response to biological drugs in pediatric inflammatory bowel disease.

Gen	RS ID	Effect	Treatment	Patient (Age)	Reference
*ATG16L1*	rs2241880	PNR	IFX	CD+UC (<21)	[98]
*IRF-AS1*	rs2188962	PNR	IFX	CD+UC (<21)	[98]
*CDKAL1*	rs6908425	PNR	IFX	CD+UC (<21)	[98]
*None*	rs762421	PNR	IFX	CD+UC (<21)	[98]
*None*	rs2395185	PNR	IFX	CD+UC (<21)	[98]
*BRWD1*	rs2836878	PNR	IFX	CD+UC (<21)	[98]
*TACR1*	rs975664	PNR	IFX	CD+UC (<21)	[98]
*TAFA4*	rs4855535	PNR	IFX	CD+UC (<21)	[98]
*None*	rs4796606	PNR	IFX	CD+UC (<21)	[98]
*PHACTR3*	rs6100556	PNR	IFX	CD+UC (<21)	[98]
*CNBD1*	rs2943177	PNR	IFX	CD+UC (<21)	[98]
*COL22A1*	rs11991611	PNR	IFX	CD+UC (<21)	[98]
*DOCK1*	rs3740543	PNR	IFX	CD+UC (<21)	[98]
*LRRC7*	rs7521532	PNR	IFX	CD+UC (<21)	[98]
*CLSTN2*	rs4605505	PNR	IFX	CD+UC (<21)	[98]
*TNFRSF21*	rs2103867	PNR	IFX	CD+UC (<21)	[98]
*PHACTR1*	rs10485363	PNR	IFX	CD+UC (<21)	[98]
*HAPLN2*	rs3795727	PNR	IFX	CD+UC (<21)	[98]
*PHACTR1*	rs6906890	PNR	IFX	CD+UC (<21)	[98]
*NLRP13*	rs302827	PNR	IFX	CD+UC (<21)	[98]
*ETV6*	rs2723829	PNR	IFX	CD+UC (<21)	[98]
*LRP1B*	rs1372256	PNR	IFX	CD+UC (<21)	[98]
*DCHS2*	rs13138970	PNR	IFX	CD+UC (<21)	[98]
*KIAA1755*	rs1205434	PNR	IFX	CD+UC (<21)	[98]
*TACR1*	rs7588326	PNR	IFX	CD+UC (<21)	[98]
*TACR1*	rs3771823	PNR	IFX	CD+UC (<21)	[98]
*ATXN1*	rs12527937	PNR	IFX	CD+UC (<21)	[98]
*KCNQ5*	rs3757105	PNR	IFX	CD+UC (<21)	[98]
*CNTN1*	rs278917	PNR	IFX	CD+UC (<21)	[98]
*HAPLN2*	rs12567958	PNR	IFX	CD+UC (<21)	[98]
*CNBD1*	rs1880473	PNR	IFX	CD+UC (<21)	[98]
*LINC00290*	rs7689941	PNR	IFX	CD+UC (<21)	[98]
*GPC3*	rs1264379	PNR	IFX	CD+UC (<21)	[98]
*TPST2*	rs3088103	PNR	IFX	CD+UC (<21)	[98]
*TRERF1*	rs4711716	PNR	IFX	CD+UC (<21)	[98]
*MGAM*	rs10464448	PNR	IFX	CD+UC (<21)	[98]
*EEPD1*	rs2540678	PNR	IFX	CD+UC (<21)	[98]
*LINC00290*	rs7659755	PNR	IFX	CD+UC (<21)	[98]
*None*	rs770389	PNR	IFX	CD+UC (<21)	[98]
*CNTN1*	rs7309734	PNR	IFX	CD+UC (<21)	[98]
*CPA6*	rs10808755	PNR	IFX	CD+UC (<21)	[98]
*RBM26*	rs1155848	PNR	IFX	CD+UC (<21)	[98]
*None*	rs1592749	PNR	IFX	CD+UC (<21)	[98]
*None*	rs765132	PNR	IFX	CD+UC (<21)	[98]
*None*	rs4707930	PNR	IFX	CD+UC (<21)	[98]
*None*	rs7905482	PNR	IFX	CD+UC (<21)	[98]
*None*	rs7059861	PNR	IFX	CD+UC (<21)	[98]
*None*	rs5975453	PNR	IFX	CD+UC (<21)	[98]
*None*	rs4077511	PNR	IFX	CD+UC (<21)	[98]
*None*	rs2825673	PNR	IFX	CD+UC (<21)	[98]
*None*	rs7003556	PNR	IFX	CD+UC (<21)	[98]
*None*	rs1243519	PNR	IFX	CD+UC (<21)	[98]
*None*	rs2044111	PNR	IFX	CD+UC (<21)	[98]
*DGKB*	rs17168564	PNR	IFX	CD+UC (<21)	[98]
*LOC105379171*	rs7726515	PNR	IFX	CD+UC (<21)	[98]
*TSPAN18*	rs835780	PNR	IFX	CD+UC (<21)	[98]
*TSPAN18*	rs835791	PNR	IFX	CD+UC (<21)	[98]
*TSPAN18*	rs7124825	PNR	IFX	CD+UC (<21)	[98]
*None*	rs9556658	PNR	IFX	CD+UC (<21)	[98]
*None*	rs1555901	PNR	IFX	CD+UC (<21)	[98]
*None*	rs4465121	PNR	IFX	CD+UC (<21)	[98]
*None*	rs10269232	PNR	IFX	CD+UC (<21)	[98]
*DGS2-AS1*	rs1667216	PNR	IFX	CD+UC (<21)	[98]
*None*	rs9404502	PNR	IFX	CD+UC (<21)	[98]
*None*	rs5977968	PNR	IFX	CD+UC (<21)	[98]
*None*	rs12937472	PNR	IFX	CD+UC (<21)	[98]
*None*	rs4301261	PNR	IFX	CD+UC (<21)	[98]
*None*	rs6529954	PNR	IFX	CD+UC (<21)	[98]
*None*	rs12559781	PNR	IFX	CD+UC (<21)	[98]
*None*	rs2825699	PNR	IFX	CD+UC (<21)	[98]
*DCDC2C*	rs11903032	PNR	IFX	CD+UC (<21)	[98]
*TLR4*	rs5030728	SubT-IFX	IFX	CD+UC (<18)	[100]
*LY96*	rs11465996	LTR	IFX, ADL	UC (<18)	[100]
		SubT-IFX	IFX	CD+UC (<18)	[100]
*TLR2*	rs1816702	SupT-IFX	IFX	CD+UC (<18)	[100]
		AB-ADL	ADL	CD+UC (<18)	[100]
*TNFRSF1B*	rs3397	LTR	IFX, ADL	CD (<18)	[100]
		SubT-ADL	ADL	CD+UC (<18)	[100]
*CD14/TMCO6*	rs2569190	AB-IFX	IFX	CD+UC (<18)	[100]
		AB-ADL	ADL	CD+UC (<18)	[100]
		Sup-IFX	IFX	CD+UC (<18)	[100]
*IL10/IL19*	rs1800872	LTR	IFX, ADL	CD (<18)	[100]
*IL17A*	rs2275913	LTR	IFX, ADL	CD (<18)	[100]
	rs10499563	LTR	IFX, ADL	CD (<18)	[100]
HLA-DQA1*05		Higher immunogenicity			[97]
*FCGR3A*	rs396991	RCR, Higher immunogenicity, Lower IFX levels	IFX	CD+UC (7–18)	[101]

PNR, primary non-response; LTR, long-term response; CD, Crohn’s disease; UC, ulcerative colitis; IFX, infliximab; ADL, adalimumab; SubT, subtherapeutic trough levels; SupT, supratherapeutic trough levels; AB, absolute trough level; RCR, reduced clinical response at the end of induction, at 22 weeks and at 52 weeks.

## Data Availability

Data sharing not applicable.
